# The British Journals: Anatomy and Physiology

**Published:** 1839-04

**Authors:** 


### III. THE BRITISH JOURNALS.

(FOR THE QUARTER ENDING FEBRUARY 28, 1839.)

### ANATOMY AND PHYSIOLOGY.

*On the Origin and Development of the Pulps and Sacs of the Human Teeth.* By JOHN GOODSIR JUNIOR, Surgeon, Anstruther.

[THIS is an elaborate and valuable paper, containing the result of much original observation, and establishing several facts in the physiology of dentition, previously but very imperfectly known. We gave some account of it in our Sixth Vol. (p. 583.) in our Report of the Newcastle Meeting of the British Association. In its present completer state we recommend it to the notice of physiologists. In addition to our former notice, we extract the brief statement of the conclusions to which careful observation of the whole process of dentition has led the author.]

*Milk Teeth.* 1. The milk teeth are formed on both sides of either jaw, in three divisions, a molar, a canine, and an incisive, in each of which dentition proceeds in an independent manner.

2. The dentition of the whole arch proceeds from behind forwards; the molar division commencing before the canine, and the latter before the incisive.

3. The dentition of each of the divisions proceeds in a contrary direction; the anterior molar appearing before the posterior, the central incisive before the lateral.

4. Two of the subordinate phenomena of dentition also obey this inverse law; the follicles closing by commencing at the median line, and proceeding backwards, and the dental groove disappearing in the same direction.

5. Dentition commences in the upper jaw, and continues in advance during the most important period of its progress. The first tooth germ which appears is that of the *superior* anterior molar, which precedes that of the *inferior* anterior molar.

The apparent exception to this law in the case of the inferior incisive has already been explained.

*Permanent Teeth.* 6. The germs of the permanent teeth, with the exception of that of the anterior molar, appear in a direction from the median line backwards.

7. The milk teeth originate, or are developed, from the mucous membrane.

8. The permanent teeth, also originating from mucous membrane, are of independent origin, and have no connexion with the milk teeth.

9. A tooth pulp and its sac must be referred to the same class of organs as the combined papilla and follicle from which a hair or feather is developed, viz. bulbs.

*Edinburgh Med. and Surg. Journal. Jan.* 1839.

*An Experimental Enquiry into the Physiological Action, the Poisonous Properties, and the Therapeutic Effects of the Hydrocyanic Acid.* By HENRY LONSDALE, M.D.

[THIS paper (which looks like an inaugural thesis, and would make a very good one,) contains a good digest of our knowledge of this important subject, and also some original experiments. The following extracts give all the more important matter embodied in it.]

1. *On the Physiological Action of Hydrocyanic Acid.*

The experiments are divided into three classes. 1. Those made with the diluted or medicinal acid of this country, which contained 3½ per cent. of anhydrous acid. 2. Those made with acid containing 12 per cent. of pure acid. 3. Those with the anhydrous acid. Twelve experiments were made with the diluted acid on dogs, cats, and rabbits.

From the above experiments (says Dr. L.) it may be safely inferred that the diluted or medicinal acid does not kill by arresting the heart's movements, or, in other words, does not produce death in the way of syncope. Since it is obvious that this acid does not exert any special influence on the heart's action, we look for some other explanation of the cause of death, which fortunately soon suggests itself on a due observance of the symptoms. That it must act on the central organs of the nervous system, the brain and spinal cord, is fully shown by the convulsive movements, vertigo, tetanic spasms, loss of sensibility and volition. And that coma is also the result of this action is readily apparent from the fact, that the respiratory acts are performed slowly and imperfectly. It is believed, then, that death takes place in the way of coma from the respiration being ultimately arrested; and although the circulation continues for some minutes scarcely unaltered, the venous blood gradually becomes impeded in its passage through the lungs, and consequently the further progress of this fluid to and from the right side of the heart is suspended.

2. Two experiments were made with the 12 per cent. acid. An aged bitch swallowed twenty drops, and ceased to breathe within the minute. A large dog formed the subject of the second experiment. An ounce was poured down his throat: the contents of the bottle were scarcely emptied, when the animal cried out and experienced convulsions, and in less than twenty-five seconds respiration had ceased. The heart of both animals contracted regularly for three minutes. Allowing the blood to escape from the *vena cava*, renewed the contractions some time after respiration had ceased, in the first experiment, so late as the fifteenth minute. In the second experiment the heart was more decidedly affected.

3. The acid I employed in the third class of experiments was made according to Gay-Lussac's method; by decomposing bicyanide of mercury with hydrochloric acid. The beak of the retort contained fused chloride of calcium and dry carbonate of lime: the receiver was kept quite cool. On Mr. Kemp's authority the acid so prepared was perfectly anhydrous. . . .

These eight experiments with the strong acid offered the same results; the symptoms bearing the closest analogy to those observed to follow the administration of the dilute acid, with the exception of its acting more energetically on the heart in these last experiments: being satisfied that the pure acid administered by the mouth, even in doses of twenty drops, though it enfeeble, does not destroy the irritability of the heart or voluntary muscles.

From a careful review of the preceding facts, I am constrained to believe that the immediate effects of the anhydrous acid are exerted on the brain and spinal cord, and that it also indirectly enfeebles, to a greater or less extent, the contractility of the heart. Cases may occasionally be met with (but I feel assured that they will be few in number) which may lead an experimenter to doubt the accuracy of my conclusions; but I think I may venture to say, that, if he performs as great a number of experiments with the substance as I have done, he will be satisfied that such instances are rare, and must be ascribed to individual idiosyncrasy.

II. *Is Hydrocyanic Acid a Cumulative Poison?*

Dr. L. answers this question in the negative.

### III. *Medico-Legal Questions.*

The examination of a medical jurist in a case of poisoning with this substance may be resolved into three questions. 1. What time elapses between the taking of the prussic acid and the first appearance of untoward symptoms? 2. Within what period does this poison prove fatal? 3. Are the symptoms induced in man by a poisonous dose of prussic acid always uniform and characteristic?

From a careful calculation of the ages, weight, &c. of several animals, and the doses administered to them as compared with man, I think it probable that a drachm of Scheele's acid would affect an ordinary adult within the minute, and a dose exceeding this, suppose three or four drachms, would exert its influence within ten or fifteen seconds. When the acid is stronger, and the quantity larger, we are pretty certain of its immediate action, and consequent annihilation of the sensorial functions.

There are records of cases of the human species poisoned by the prussic acid where death occurred as early as the second minute, and as late as the forty-fifth minute. Should proper measures be adopted for the recovery of those poisoned, it seems probable that those who survive the fifteenth minute may recover.

The loss of consciousness and voluntary motion, the slow, deep, and heaving respiratory movements, whilst the circulation gradually becomes enfeebled, the fixed and insensible iris, in short, all the characteristics of apoplexy, were observed in the lower animals, and the same were observed in the physician at Rennes. It is far from improbable that a person may have a dose of prussic acid given him, so proportioned as to induce a simulated apoplexy, which, if the medical man was unaware of the cause, might be mistaken for the real disease. For it must be remembered that when doses not rapidly fatal are administered, the convulsions and tetanic spasms soon subside, and the animal lies in the apoplectic state for some time. So far then there is some difficulty in the way, and a knowledge of the general symptoms would be inadequate. But there is one circumstance which has received no attention from medico-legal writers, and that is the exhalation by the breath of the acid vapour. This has been observed in the human species, and numerous animals experimented upon by me, as already mentioned, and certainly would be sufficiently characteristic of the poison having been taken.

With the view of ascertaining the duration of the odour after death, I caused several animals which had been poisoned by the acid to be kept in a room at the temperature of 50° Fahrenheit, during seventeen hours of the day, and others to be buried in light garden soil six inches below the surface. The results of these experiments (fifteen in number), which were tested by at least three individuals in each case, lead me to expect the odour as late as the eighth or ninth day after death, even where life is prolonged to the eighth minute after a dose of the hydrocyanic acid.

My chemical examination of the blood, brain, and contents of the stomach, according to the method recommended by Leuret and Lassaigne, leads to the following conclusion: that from the fourth to the eighth day after death, although the odour is sufficiently strong so as not to be mistaken, yet careful distillation and the usual tests fail to prove the presence of the acid in animals which have been kept in the apartment where they were poisoned.

### IV. *On the Treatment of Poisoning with Hydrocyanic Acid.*

[After briefly noticing the more ordinary remedies, Dr. L. adds:]

Looking at the mode in which death takes place,—the continuation of the heart's action for a short time after the respiration has ceased,—the gradually increasing distension of the right side of the heart by venous blood, and then the final arrestment of the movements of this organ; the renewal of the contractions of the pulmonic heart after the withdrawal of a small quantity of blood from its auricle, are all circumstances of the utmost importance, as suggesting the principles of treatment. It has already been stated that the acid, although having a direct influence on the central organs of the nervous system, also affects the contractility of the heart, and that this effect on this organ varied from a slight diminution to complete suspension of its contractility, according to the amount of the dose and other circumstances. In those cases in which coma is induced, without much, if any, diminution in the contractile power of the heart, the blood notwithstanding becomes congested in the right side of that organ, from the imperfect manner in which it is transmitted through the lungs, as occurs in all cases where the respiratory movements are imperfectly performed: bleeding from the jugular, by relieving the engorged state of the right side of the heart, may be expected to favour the remedial agent, such as dashing cold water upon the face, applying ammonia to the nostrils, &c. employed to revive the patient and cause him to breathe. It also appears that when the effects of the acid upon the heart are transitory, and produce only a temporary diminution of its contractility, the right side of the heart becomes engorged during the partial suspension of its movements, and that when it would renew its usual contractions, it is prevented from doing so by the mechanical distension of its walls. By opening the jugular, in these circumstances, we may hope to relieve the engorgement of the right side of the heart, and thus materially favour the renewal of its contractions.

[To put these theoretical opinions to the test, several experiments were performed by Dr. Lonsdale and his friends, on dogs, and with very satisfactory results.]

From these experiments (he says) I feel no hesitation in saying, what might indeed be inferred *à priori*, from an acquaintance with the physiological action of the poison, that bleeding from the jugular vein is of essential service in the treatment of poisoning with hydrocyanic acid. It evidently acts by unloading the congested cavities of the right side of the heart, which enables this viscus to renew its contractions until the coma subsides.

V. *Therapeutical Properties of the Hydrocyanic Acid.*

This section contains nothing new.

VI. *In what manner does Hydrocyanic Acid act as a Poison?*

In this section the author briefly notices the views of different authors, but arrives at no definite conclusion. I am willing to believe (he says) that, although the hydrocyanic acid is absorbed and carried into the circulation, its *modus operandi* upon the sentient extremities of nerves seems most probable.

*Edinburgh Med. and Surg. Journal. Jan.* 1839.

---

### *Of a Peculiarity of Structure occasionally occurring in the Basilar Artery of Man.* By John Davy, M.D., F.R.S.

Besides those peculiarities of structure of the basilar artery which are well known, there is one of not uncommon occurrence which, to the best of my knowledge, has not hitherto been noticed by any anatomist. It is a band in the interior of the vessel, attached to its side, and consequently intersecting it. It varies both in its dimensions and situation. I have most frequently found it near the junction of the vertebral arteries; very seldom near the commencement of the circle of Willis. Sometimes the band perfectly, at other times only partially, intersects the vessel. Sometimes I have seen it not more than one line thick, occasionally two or three lines. Its appearance, as regards its nature, has always been similar and most analogous to a fibrous structure. In every instance, I apprehend, it may be considered as congenital, and not the effect of disease.

Whether this peculiarity of structure has a decided use I am not prepared to say. In each instance in which a band or fibre presents itself, support is afforded, and additional strength is imparted. The band I have described, as occasionally occurring in the basilar artery, must necessarily have this effect. It was in the month of June last year that my attention was first attracted to the subject. Since that time I have availed myself of every opportunity that has offered to examine the basilar artery. In ninety-eight post-mortem examinations at which I have been present, made in the General Hospital at Fort Pitt, during the period, I have met with it in seventeen instances. In nine, death was owing to pulmonary consumption; in two, to malignant tumour; in the remainder, in each instance to a different disease. The ages of the individuals varied from 19 to 59; two were 25; two 35. In point of age there was no other accordance amongst them.

*Edinburgh Med. and Surg. Journal. Jan.* 1839.

---

*On the relative [constitutional] Prevalence of certain Organic Diseases.* By JOHN DAVY, M.D., F.R.S.

I AM disposed to believe that, were the pathological anatomist engaged in extensive and minute research, to institute a series of observations on organic changes analogous to that which Sydenham conducted in ordinary maladies, he might arrive at the conclusion, that there are organic constitutions prevalent at times, not less than atmospheric, and which (however produced) may be as much concerned in the origin of chronic disease, as the atmospheric influences are in the acute.

I shall give, in a tabular form, some of the results of my experience bearing on this subject, drawn from my notes of the various post-mortem examinations which I have attended during a period of nearly eighteen years, namely, from May 1821, when I commenced the practice of making a note of every fatal case in which there was an examination of the body after death. My experience was chiefly confined to our military hospitals; indeed, at home it was entirely so restricted. On foreign stations it extended to the native population, especially in Malta, where the civil hospital offers an ample field for research.

| Stations. | Years. | No. of bodies examined. | Instances of tubercles in the lungs. | Of ulceration of larynx with ditto. | Of ulceration of the larynx without ditto. | Of Pneuma-thorax. | Of varicose lacteals. | Of apertures in fossa ovalis. |
|---|---|---|---|---|---|---|---|---|
| England, Fort Pitt | 1821 | 29 | 11 | | | | | 1 |
| | 1822 | 61 | 33 | 19 | | | 6 | 10 |
| | 1823 | 40 | 20 | 7 | | 1 | | 2 |
| Ionian Islands | 1824 | 10 | | 2 | 1 | | | |
| | | 18 | 5 | | 1 | | | 2 |
| | 1825 | 36 | 2 | 2 | | | | 1 |
| | 1826 | 9 | 3 | | | | | |
| | 1827 | 33 | 8 | 1 | | | | 3 |
| Malta | 1828 | 6 | | | | | | |
| | | 32 | 5 | 1 | | | 1 | 5 |
| | 1829 | 19 | 4 | 1 | | | | 3 |
| | 1830 | 41 | 5 | 1 | 1 | | | 9 |
| | 1831 | 54 | 19 | 3 | | 1 | 2 | 8 |
| | 1832 | 55 | 8 | 1 | | 3 | | 7 |
| | 1833 | 49 | 17 | 9 | | 1 | | 5 |
| | 1834 | 57 | 15 | 5 | | 3 | 1 | 5 |
| England, Fort Pitt | 1835 | 10 | 4 | 1 | | | | |
| | | 31 | 20 | 2 | | 2 | | 1 |
| | 1836 | 72 | 49 | 18 | | 1 | | 8 |
| | 1837 | 81 | 50 | 15 | 1 | 5 | | 8 |
| | 1838 | 43 | 45 | 11 | | 1 | | 3 |
| Total* | 18 | 786 | 308 | 108 | 4 | 19 | 10 | 82 |
| | | | 323 | 99 | | 18 | | 81 |

On the results contained in this table, I shall restrict myself at present to a very few remarks.

The very large proportional number of cases in which tubercles were found in the lungs, viz. 39 per cent. of the whole, may excite surprise. I must confess it

* We have printed the totals as given in the Ed. Journal; but there are errors somewhere. If the number of individuals are correct, the totals should be as we have given them in the smaller figures.

had that effect on my mind, and the more so, as no doubtful instances were admitted. I rigorously rejected every example not coming under the denomination of the consumptive tubercle; that is, a tubercle, albuminous in composition; admitting of induration by boiling, as pointed out by Dr. Abercrombie, and of softening in the progress of disease, giving rise to vomicæ and excavations in the lungs. The melanotic tubercle was excluded, as also certain concretions more or less resembling tubercles, whether consisting principally of phosphate of lime and the other materials of bone, or of a nature approaching to cartilage. I may also remark, that no cases were admitted as supposed instances of tubercles, from the presence merely of cavities in the lungs. Cavities existing unaccompanied by tubercles were inferred to be examples of pulmonary abscess, of which several instances occurred.

No doubt the astounding frequency of tubercles recorded in the table, is partly owing to the description of cases sent to the General Hospital at Fort Pitt, where a considerable proportion of the whole mortality under observation occurred. But, making the most ample allowance on this account, I apprehend the conclusion is unavoidable, that the existence of tubercle is far more frequent than is commonly supposed, and the reported deaths from phthisis would indicate.

Of the four instances of ulceration of larynx unaccompanied by tubercles, the first was complicated with empyema and purulent effusion within the pericardium, to the extent of three pounds and a half; the second was associated with melanotic tubercles in the lungs; and the third and fourth were connected with smallpox.

All the instances of pneumathorax, with the exception of one, occurred in cases of tubercular phthisis, and originated in a communication of a valvular kind being established between the pleura and a bronchial tube by ulceration, commonly through the medium of a cavity. In the one exception, a similar communication was detected, the consequence of a partial destruction of lung from an abscess in the liver, penetrating and bursting into the lung through the diaphragm.

Of the large number of examples of aperture in the fossa ovalis, all were oblique, with the exception of three. Of the three direct, one was sufficiently large to admit the fore-finger, and two to admit the end of the little-finger. In neither instance was there the slightest appearance of the morbus cæruleus. The subject of the first-mentioned and most remarkable example was an old soldier, who for many years enjoyed excellent health. In these cases, it appears inevitable that there must have been an admixture of venous and arterial blood in the auricles. In the examples of the oblique passage, the probability is, that no blood flowed from one auricle into the other. In one instance, in which the oblique aperture was sufficiently large to receive the end of the little-finger, the right cavities of the heart were found distended with coagulated blood, and the left empty.

*Edinburgh Med. and Surg. Journal. Jan.* 1839.

